# Role of Farnesoid X Receptor in the Pathogenesis of Respiratory Diseases

**DOI:** 10.1155/2020/9137251

**Published:** 2020-11-26

**Authors:** Jin-nan Wu, Jian-rong Chen, Jin-liang Chen

**Affiliations:** ^1^Postgraduate in Respiratory Medicine, Affiliated Hospital 2 of Nantong University, Nantong, China; ^2^Department of Respiratory Medicine, Affiliated Hospital 2 of Nantong University, Nantong, China

## Abstract

Farnesoid X receptor (FXR) is a bile acid receptor encoded by the Nr1h4 gene. FXR plays an important role in maintaining the stability of the internal environment and the integrity of many organs, including the liver and intestines. The expression of FXR in nondigestible tissues other than in the liver and small intestine is known as the expression of “nonclassical” bile acid target organs, such as blood vessels and lungs. In recent years, several studies have shown that FXR is widely involved in the pathogenesis of various respiratory diseases, such as chronic obstructive pulmonary disease, bronchial asthma, and idiopathic pulmonary fibrosis. Moreover, a number of works have confirmed that FXR can regulate the bile acid metabolism in the body and exert its anti-inflammatory and antifibrotic effects in the airways and lungs. In addition, FXR may be used as a potential therapeutic target for some respiratory diseases. For example, FXR can regulate the tumor microenvironment by regulating the balance of inflammatory and immune responses in the body to promote the occurrence and development of non-small-cell lung cancer (NSCLC), thereby being considered a potential target for immunotherapy of NSCLC. In this article, we provide an overview of the internal relationship between FXR and respiratory diseases to track the progress that has been achieved thus far in this direction and suggest potential therapeutic prospects of FXR in respiratory diseases.

## 1. Introduction

Farnesoid X receptor (FXR) is a ligand-activated transcription factor encoded by the Nr1h4 gene; this transcription factor belongs to the bile acid receptor of the nuclear receptor superfamily [1]. FXR plays a vital role in maintaining the stability of the internal environment and the integrity of various organs, including the liver and intestines. A large number of studies have demonstrated that FXR plays an important role in the metabolism of bile acid [2], cholesterol [3], lipids [4], and glucose [5] and the regulation of intestinal flora [6]. It is also widely involved in the occurrence and development of cholestasis [7], alcoholic liver disease [8], primary biliary cirrhosis [9], inflammatory bowel disease [10], atherosclerosis [11], and even gastrointestinal tract and other malignant tumors [12]. In addition, FXR can be used as a potential therapeutic target for several diseases [13, 14]. Recent studies have suggested that FXR may play a considerable role in the physiological and pathological aspects of the respiratory system [15–17]. In this article, we provide an overview of the internal relationship between FXR and respiratory diseases to track the progress that has been achieved thus far in this direction and suggest potential therapeutic prospects of FXR in respiratory diseases.

## 2. Biological Characteristics of FXR

### 2.1. Distribution and Structure of FXR

FXR was isolated by Forman for the first time in a rat liver cDNA library [18]. FXR was initially considered as a simple orphan nuclear receptor. However, bile acid metabolites were observed to have the ability to fully bind to FXR at the physiological level. Hence, FXR was finally identified as a bile acid receptor. FXR is presently considered as a kind of bioreceptor for bile acid receptors and synthesis that fully participates in the regulation of bile acid metabolism. FXR plays an important role in maintaining the stability of the internal environment and the integrity of various organs, including the liver and intestines [19, 20].

FXR has two known genotypes, namely, FXR*α* (NR1H4) and FXR*β* (NR1H5). Since its discovery, the FXR gene has been successfully cloned in numerous species, including humans, rats, and mice. The single FXR*α* gene in humans and rodents encodes four different isoforms, namely, FXR*α*1, FXR*α*2, FXR*α*3, and FXR*α*4, which are caused by different promoters and RNA splicing, and their expression is tissue specific [21]. However, FXR*β* is expressed as a pseudogene in human and primates, and its mechanism of action remains unclear [22, 23]. Therefore, FXR is used to express FXR*α* in this review. The human FXR gene is located on chromosome 12 (12q23.1), which is mainly expressed in various digestible tissues, including the liver [24] and small intestine [25]. In addition, the expression of FXR in nondigestible tissues other than in the liver and small intestine is known as the expression of “nonclassical” bile acid target organs, such as kidneys [26], adrenal glands [27], lung tissue [13], and blood vessels [28].

As an orphan nuclear receptor, FXR has a typical nuclear receptor structure [29]. Its structure includes highly conserved carboxy-terminal ligand-binding domain (LBD), amino-terminal DNA-binding domain (DBD), low conserved amino-terminal ligand-independent transcription activation functional domain (AF-1), carboxy-terminal ligand-dependent transcription activation functional area (AF-2), and hinge area connecting LBD and DBD. FXR is a ligand-activated transcription factor classified as a nuclear bile acid receptor. Under the action of a ligand, the FXR that is activated by an agonist (such as bile acid) enters the nucleus of the cells and then combines with retinoid *X* receptor to form a heterodimer, which can be bound to a specific FXR response elements and regulate the expression of various target genes by changing the structural conformation of FXR [30, 31].

### 2.2. Function of FXR

FXR was first widely recognized for regulating the bile acid pathway in the liver and intestines. Previous studies have demonstrated that FXR regulates the bile acid synthesis through two main pathways [32–34]. In the classical pathway, CYP7A1 is a liver-specific microsomal cytochrome P450 enzyme, which is a rate-limiting enzyme in the classic pathway of bile acid synthesis. CYP7A1 is only expressed in the human liver and can catalyze the 7*α* hydroxylation of cholesterol. In the alternative pathway, CYP27A1 initiates an alternative pathway of bile acid synthesis, in which cholesterol is oxidized to form chenodeoxycholic acid. The main physiological function of FXR is to regulate the conversion of cholesterol into bile acid, which is the basis for the body to participate in other biochemical reactions.

Recent studies have reported that FXR plays an important role in regulating lipid and glucose metabolism [4, 5]. Moreover, FXR plays a vital role in preventing disorder infections of intestinal flora and formation of biliary calculus [6, 7]. FXR activation exhibits beneficial effects on various metabolic diseases, including fatty liver [35], type 2 diabetes [36], and hyperlipidemia [37]. Accumulating evidence supports the observation that FXR agonists are beneficial to the regulation of enterohepatic circulation [38], liver regeneration [39], and occurrence and development of metabolic tumors, such as liver cancer [40]. Moreover, FXR agonists prevent the development of atherosclerosis [11] and kidney diseases [41], and they are valuable for the treatment of diabetes, hyperlipidemia, and obesity [42, 43]. These properties indicate that FXR activation has a systemic effect and may be a potential strategy for the treatment of a variety of disorders.

## 3. FXR and Respiratory Diseases

Increasing evidence shows that the expression of FXR in “nonclassical” bile acid target tissues, such as blood vessels and lungs [15, 28], is equivalently important. In the vasculature, FXR regulates cholesterol transport and vascular tension by regulating its own expression [44, 45]. Hendrick et al. confirmed the presence of FXR in human airway epithelial cell lines and pulmonary vascular endothelial cell lines [46]. Zhang et al. [47] established that FXR could inhibit lipopolysaccharide- (LPS-) induced acute lung inflammation injury, as well as regulate and improve lung regeneration in animal experimental models. Several studies have reported that FXR agonists, such as 6-ethylchenodeoxycholic acid (OCA), play an anti-inflammatory and antifibrotic role in lung tissues, and FXR can be used as a new target for the treatment of certain lung diseases [13, 48]. This review provides an overview of these studies and then summarizes the latest progress in FXR research on respiratory diseases.

### 3.1. Chronic Obstructive Pulmonary Disease

Chronic obstructive pulmonary disease (COPD) is a common and frequently occurring respiratory disease, with high morbidity and mortality. COPD is characterized by continuous and mostly progressive airflow limitation mainly due to abnormal airway remodeling and chronic inflammation. Lesions in small airways, such as small airway inflammation, fibrous tissue formation, and lumen mucus plugs, can lead to increased peripheral airway resistance in patients with COPD [49]. Although the mechanism of airway remodeling in COPD has not been fully clarified, the process involves epithelial-mesenchymal transition (EMT) [50], the development of which is related to the severity of airway restriction [51]. In addition, cyclooxygenase 2 (COX-2) is a key enzyme involved in converting arachidonic acid to prostaglandin E2, which is an important factor leading to the airway inflammation of COPD. In this manner, the expression of COX-2 is also related to the severity of airflow limitation [52, 53].

Chen et al. [54] detected FXR expression in human small airway epithelial cells and also in the rat COPD lung model. They found that FXR could promote bile acid-induced EMT in alveolar epithelial cell lines, suggesting that the overexpression of FXR may lead to airflow limitation in patients with COPD by promoting EMT in the small airways. Furthermore, they observed that COX-2 is overexpressed in the small pulmonary airways of COPD, and bile acid inhalation may promote airway inflammation through FXR-mediated COX-2, which in turn results in continuous airflow limitation of COPD. However, FXR could also inhibit LPS-induced acute lung inflammation [47]. This contradictory effect of FXR may be related to various causes of respiratory diseases, a research direction that requires further investigation. Nevertheless, FXR does participate in the pathogenesis of COPD.

### 3.2. Asthma

Asthma is among the most common chronic diseases worldwide. It is a chronic inflammatory disease of the airways involving multiple types of cells and cell components [55]. It is characterized by airway inflammation, hyperresponsiveness, and remodeling. On the one hand, chronic airway inflammation of asthma is due to the activated helper Th2 cells that produce interleukins, such as interleukin-4 (IL-4), interleukin-5 (IL-5), and interleukin-13 (IL-13), to activate B lymphocytes, synthesize specific immunoglobulin E, and activate mast cells and eosinophils to mediate the synthesis and release of a variety of active mediators [56]. On the other hand, the production of cytokines, such as interleukins, by activated helper Th2 cells can directly activate mast cells, eosinophils, and alveolar macrophages, which in turn can secrete a variety of inflammatory mediators and cytokines, such as histamine, leukotrienes, prostaglandins, and transforming growth factors [57, 58]. In addition, nuclear factor-kappaB (NF-*κ*B), as an important nuclear transcription factor in the cell, is widely involved in the inflammatory and immune responses, can regulate cell apoptosis and stress response, and plays an important role in the pathogenesis of asthma [59, 60].

Shaik et al. [61] found that FXR mRNA and protein can be expressed in mouse lung tissues and in rat and human lung endothelial cells, indicating that FXR is involved in normal physiological responses in lung tissues. In the constructed ovalbumin-induced acute rat asthma model, they found that the anti-inflammatory effect of FXR is achieved by blocking the infiltration of inflammatory cells into normal lung tissues, thereby inhibiting the secretion of IL-4, IL-5, and IL-13 by activated helper Th2 cells and that of tumor necrosis factor-*α* (TNF-*α*) by activated alveolar macrophages. Moreover, they noted that FXR may partially reduce airway inflammation in asthma by antagonizing NF-*κ*B signaling and target gene expression in vivo.

### 3.3. Idiopathic Pulmonary Fibrosis

Idiopathic pulmonary fibrosis (IPF) is a chronic, progressive, and fibrotic interstitial pneumonia [62]. It has typical histopathological features or radiographic images of common interstitial pneumonia. The pathogenesis of IPF is not completely clear. IPF may be the result of the interaction between genetic and environmental factors. From the perspective of pathophysiology, IPF is presently understood to originate from abnormal repair of alveolar epithelium after repeated minor injury [63]. Repeated minor injury leads to the apoptosis of alveolar epithelium, and abnormal activation of epithelium induces the proliferation of intrinsic fibroblasts which stimulate the development of EMT and the differentiation of fibroblasts into myofibroblasts. These processes in turn lead to progressive pulmonary interstitial fibrosis and airway stiffness that can cause breathing difficulties and eventually result in respiratory failure. EMT, inflammatory processes, and collagen deposition are considered as important mechanisms in the pathogenesis of IPF [64, 65].

EMT is a process, in which fully differentiated epithelial cells gradually transform into mesenchymal phenotypes that involves the activation of transforming growth factor-*β*1 (TGF-*β*1), connective tissue growth factor, epidermal growth factor, and platelet-derived growth factor subunit A [66]. The common feature of these pathways is that they have the ability to promote the activation of “major transcription factors,” such as zinc finger transcription factors 1 (SNAI1) and zinc finger transcription factors 2 (SNAI2), which are responsible for initiating the EMT program in epithelial cells [67, 68].

FXR is not only located in alveolar epithelial type I cells (AECIs) but also in alveolar epithelial type II cells (ATECIIs) [69]. ATII cells are multifunctional cells that synthesize and secrete lung surfactants and can participate in the immune response by producing AECIs [70]. In addition, the expression of FXR in ATECIIs is regarded as the main source of mesenchymal expansion in pulmonary fibrosis [71]. Comeglio et al. [13, 72] reported that TGF-*β*1, SNAI1, and SNAI2 expression levels substantially increase in the constructed rat model of pulmonary fibrosis, whereas, with the participation of FXR agonists (such as OCA), their expression levels are restored to those of the control group, thereby inhibiting the development of EMT. Several studies have proposed that IL-6 is a key factor that mediates the proliferation of fibroblasts driven by TGF-*β*1 in the lungs likely by converting acute inflammation of the lungs to a more chronic fibrotic state [73]. Comeglio et al. confirmed that OCA could considerably reduce the production of proinflammatory cytokines (i.e., IL-1*β*, IL-6, and TNF-*α*) in the IPF rat model and could also regulate the proportion of matrix metalloproteinases (MMP) and their inhibitors (tissue inhibitor of metalloproteinases, TIMP), thereby alleviating the symptoms of pulmonary fibrosis. Therefore, FXR inhibits the occurrence of IPF mainly via two avenues: it inhibits the production of proinflammatory cytokines (such as IL-1*β*, IL-6, and TNF-*α*), that is, inhibiting the inflammatory stage of pulmonary fibrosis; it suppresses the expression of TGF-*β*1/SNAI1 and SNAI2 to inhibit the development of EMT and regulates the proportion of MMPs/TIMPs to reduce the situation of pulmonary fibrosis itself.

### 3.4. Pulmonary Hypertension

Pulmonary hypertension (PAH) is a chronic progressive disease with abnormally elevated pulmonary artery pressure that is characterized by increased pulmonary vascular resistance, leading to right heart failure and eventually death [74]. The key pathological change in PAH is the remodeling of small pulmonary arteries. This remodeling is characterized by thickening of the intima, media, and adventitia of small arteries, leading to the gradual narrowing of small pulmonary vessels, gradual increase in vascular resistance, and induction of adaptive right ventricular hypertrophy. The strain of constant pressure overload in the small arteries will eventually result in right heart failure [75]. Vascular remodeling in PAH is a complex and multifactorial process that involves not only perivascular inflammation, EMT, and pulmonary fibrosis but also impairs vasodilatory function mediated by pulmonary vascular endothelium [13, 76, 77].

Several studies have observed that FXR exists not only in pulmonary vascular endothelial cells but also in epithelial cells [69, 78]. FXR plays an important role in maintaining lung function by inhibiting lung inflammatory response, producing lung surfactants, and promoting alveolar repair to resist lung injury. With regard to PAH [48, 79], FXR can inhibit the expression of the proinflammatory factor IL-6 and monocyte chemotactic protein-1 (MCP-1), as well as inhibit the NF-*κ*B-mediated inflammatory response during PAH progression. Furthermore, FXR can inhibit the expression of TGF-*β*1 to inhibit EMT and pulmonary fibrosis, thereby delaying the development of PAH. In addition, the bone morphogenetic protein/bone morphogenetic protein receptor (BMP/BMPR) system plays a vital role in PAH. The lack of BMPR expression in pulmonary vascular endothelial cells can result in the increased secretion of intracellular growth factors and IL-6, thereby promoting the occurrence of TGF-*β*1-induced EMT, thus providing a circular response that aggravates the perivascular inflammation, EMT, and pulmonary vascular remodeling. FXR promotes the expression of BMP2/BMPR1A in pulmonary vascular endothelial cells, thereby improving the progress of PAH. Finally, FXR can regulate the balance of relaxation and contraction in smooth muscle cells by reducing the conduction of the NO-sGC-cGMP signaling pathway, which in turn normalizes the function of pulmonary vascular endothelium and thus inhibits the progression of PAH.

### 3.5. Acute Lung Injury and Acute Respiratory Distress Syndrome

Acute lung injury (ALI) and its more serious form, namely, acute respiratory distress syndrome (ARDS), are common diseases worldwide that may lead to acute respiratory failure and death [80]. The essence of ALI/ARDS is a variety of inflammatory cells, and their release of inflammatory factors and cytokines indirectly mediates lung inflammatory response, which can be considered as the pulmonary manifestation of systemic inflammatory response syndrome [81].

Several studies have confirmed that FXR mainly plays an important role in inhibiting lung inflammation and promoting lung regeneration in ALI/ARDS. On the one hand, FXR inhibits the release of proinflammatory cytokines (IL-1*β* and TNF-*α*) and chemokines (CXCL1 and MCP-1) and partially upregulates the expression of anti-inflammatory cytokines (IL-10) in the lungs to reduce the inflammatory response of ALI/ARDS. ALI/ARDS induces the release of proinflammatory cytokines and chemokines by activating the NF-*κ*B signaling pathway, whereas FXR inhibits the NF-*κ*B signaling pathway and also hinders MAPK and PI3K/Akt signaling pathways. In turn, FXR partially reduces the pulmonary inflammatory response of ALI/ARDS [82]. Notwithstanding, FXR activation inhibits the expression of P-selectin and induces the expression of the gene Foxm1b, thereby improving the permeability of the lungs. Moreover, FXR activation inhibits the “chemotactic movement” of leukocytes from the peripheral circulation into the inflammatory tissues of the lungs. Furthermore, FXR activation stimulates the proliferation of endothelial cells to help restore the integrity of the pulmonary vascular endothelial barrier, thereby promoting the lung repair to inhibit ALI/ARDS disease progression [47].

### 3.6. Non-Small-Cell Lung Cancer

Lung cancer is the leading cause of cancer-related deaths worldwide with the highest mortality rate among all malignant tumors [83]. Non-small-cell lung cancer (NSCLC) accounts for about 85% of all lung cancer cases [84]. A large number of studies have observed that numerous molecular changes and specific gene expressions are related to the occurrence and development of NSCLC [85, 86]. Tumor cells can reshape the tumor microenvironment that will further affect the behavior and state of tumor cells [87, 88]. The human lungs are susceptible to various poisons and pathogens. Consequently, the human lungs are prone to chronic damage and inflammation that form an inflammatory tumor microenvironment, thereby resulting in the occurrence of NSCLC [89].

FXR is regarded as a regulator of inflammation and immune response in immune-mediated disease subpopulations [47, 90]. As a ligand-activated transcription factor, FXR can control the transcription of target genes by binding to FXR response elements (FXRE) [91]. Several studies have reported that, compared with that of healthy controls, the FXR of patients with NSCLC is markedly increased and its expression level is positively correlated with poor clinical results [92]. FXR can be recruited to the promoter of CCND1 in NSCLC cells and activate its transcription. CCND1 transcription can shorten the G1/S phase transition process in the cell cycle by upregulating the transcriptional expression of cyclin D1 and then promote the growth of NSCLC cells. FXR may also promote the development of NSCLC by regulating the tumor microenvironment, especially its immunological characteristics. In the NSCLC cell model, You et al. [93] found that knocking down FXR increases the expression of PD-L1, whereas the overexpression of FXR induces the downregulation of PD-L1 expression in NSCLC cells. These results indicated that FXR inhibits the expression of PD-L1 in NSCLC cells by binding to the putative FXRE in the PD-L1 promoter. This promoter could be used as a potential target for immunotherapy of FXR^high^PD-L1^low^ NSCLC. In summary, FXR plays a carcinogenic role in the progression of NSCLC.

## 4. Conclusion

As a multifunctional nuclear receptor, FXR can participate in the immune regulation and inflammation in the body, so it is highly expressed in the respiratory system with immune defense and prone to inflammation, such as bronchial epithelial cells, alveolar epithelial cells, alveolar macrophages, and pulmonary vascular endothelial cells. As a bile acid-activated nuclear receptor, FXR can bind to the FXRE and mediate anti-inflammatory and antifibrosis responses in the respiratory system by inhibiting the expression of proinflammatory cytokines and chemokines, texpression of COX-2 in the airways, toccurrence of EMT, and conduction of NF-*κ*B, MAPKs, and PI3K/Akt signaling pathways. In addition, FXR can regulate the tumor microenvironment by regulating the balance of inflammatory and immune responses in the body to promote the occurrence and development of NSCLC. Thus, it can be a potential target for immunotherapy of NSCLC.

In summary, FXR is expressed in the respiratory system and plays an important pathophysiological function in respiratory diseases. FXR regulates the bile acid metabolism in the body and also exerts its anti-inflammatory and antifibrotic effects in the airways and the lungs. Further research on the role of FXR in the pathogenesis of various respiratory diseases would aid in developing this receptor as an indicator of the progression of these diseases and as their potential therapeutic target. It is believed that FXR can play a pivotal role in respiratory diseases in the near future.

## Data Availability

No data were used to support this study.

## Abbreviations

FXR: Farnesoid X receptor
LBD: Ligand-binding domain
DBD: DNA-binding domain
LPS: Lipopolysaccharide
COPD: Chronic obstructive pulmonary disease
EMT: Epithelial-mesenchymal transition
COX-2: Cyclooxygenase 2
IL-4: Interleukin-4
IL-5: Interleukin-5
IL-13: Interleukin-13
NF-*κ*B: Nuclear factor-kappaB
TNF-*α*: Tumor necrosis factor-*α*
IPF: Idiopathic pulmonary fibrosis
TGF-*β*1: Transforming growth factor-*β*1
SNAI1: Zinc finger transcription factors 1
SNAI2: Zinc finger transcription factors 2
AECIs: Alveolar epithelial type I cells
ATECIIs: Alveolar epithelial type II cells
MMP: Matrix metalloproteinases
TIMP: Tissue inhibitor of metalloproteinases
PAH: Pulmonary hypertension
MCP-1: Monocyte chemotactic protein-1
BMP: Bone morphogenetic protein
BMPR: Bone morphogenetic protein receptor
ALI: Acute lung injury
ARDS: Acute respiratory distress syndrome
NSCLC: Non-small-cell lung cancer
FXRE: FXR response elements.
